# Chemical Composition and Antimicrobial Activity of Essential Oil from *Phytolacca dodecandra* Collected in Ethiopia

**DOI:** 10.3390/molecules24020342

**Published:** 2019-01-18

**Authors:** Wondwosen Abebe Matebie, Wanchang Zhang, Guangbo Xie

**Affiliations:** 1 School of Life Science and Technology, University of Electronic Science and Technology of China, Chengdu 610054, China; wond3abebe@gmail.com (W.A.M.); spadezs@163.com (W.Z.); 2 Department of Chemical Engineering, Hawassa University Institute of Technology, Hawassa P.O. Box 05, Ethiopia; 3 Center for Informational Biology, University of Electronic Science and Technology of China, Chengdu 610054, China

**Keywords:** *Phytolacca dodecandra*, essential oil, HS-SPME, chemical composition, antimicrobial activity

## Abstract

The essential oil from *Phytolacca dodecandra*, a traditional herb of Ethiopia, has been studied, including the chemical composition and antimicrobial activity. The difference between four *P. dodecandra* samples (P-1–P-4), which differed in gender or location, has also been analyzed. The essential oils were obtained by steam distillation, while the aromas were extracted by head space solid-phase microextraction (HS-SPME) and both were analyzed by gas chromatography- mass spectrometry (GC-MS). The oils’ antimicrobial activities were evaluated by the microdilution method against *Escherichia coli*, *Staphylococcus aureus*, *Bacillus subtilis* and *Candida albicans*. Ninety one components, representing 88.37 to 94.01% of the aromas, were identified. The compositions of the aromas of four samples are mainly dominated by aldehydes and ketones: 2-nonanone (1.80–30.80%), benzaldehyde (4.99–25.99%), and sulcatone (2.34–5.87%). Sixty components representing 64.61 to 69.64% of the oils were identified, and phytone (3.04–21.23%), phytol (4.11–26.29%) and palmitic acid (1.49–23.87%) are the major compounds. No obvious antimicrobial activity was observed for all the four essential oils.

## 1. Introduction

Traditional medicine has been practiced virtually in all cultures, and it has expanded globally and gained popularity [1]. In Ethiopia, the knowledge of traditional medicine has been transferred from one generation to another, and about 80% of the Ethiopian population is still dependent on traditional medicine, especially on medicinal plants [2,3].

Essential oils are complex mixtures of volatile substances generally present at low concentrations, and they are important components used for their flavor and fragrances in food, pharmaceutical and perfumery industries [4].

*Phytolacca dodecandra* L′Hérit (Phytolaccaceae), called ‘endod’ in Ethiopia, is a robust perennial climber or scrambling shrub [5,6]. It was famous in Africa for its molluscicidal activity, which can be used to block the transmission of schistosomiasis [7]. The ethnobotanical data on the traditional use of *P. dodecandra* showed its ability for treatment of ascariasis, gonorrhea, malaria, rabies, jaundice, eczema, and it was also used as abortifacient [8,9,10]. According to our literature survey, many phytochemical studies on this plant have been performed, and a type of triterpenoid saponins, which had the molluscicidal activities and were named lemmatoxins after Aklilu Lemma, an Ethiopian scientist, were the major compounds isolated [11,12,13,14,15,16,17,18,19,20]. However, no research on the essential oil has been reported. In the current study, we collected four samples (P-1–P-4) of *P. dodecandra* in Ethiopia, that differed in gender or location. Therefore, the present study will focus on the chemical composition of aromas and essential oils from this important Ethiopian medicinal plant, and the antimicrobial properties of essential oils will also be studied.

## 2. Results

The essential oils from *P. dodecandra* leaves were yellowish with a yield of 0.014% (*w*/*w*) for sample P-1, 0.038% (*w*/*w*) for sample P-2, 0.016% (*w*/*w*) for sample P-3 and 0.01% (*w*/*w*) for sample P-4. The aroma and essential oils compositions are listed in Table 1.

A total of ninety-one constituents were detected from all the four aromas samples, and only six components: sulcatone, benzaldehyde, β-ionone, β-ionone epoxide, phytone and dihydro- actinidiolide were common to all of them. This means that the four plant samples are widely different. Thirty compounds were identified from the aromas of sample P-1 (Arebi endod), comprised 93.92% of the total aromas, with predominance of aldehydes and ketones (49.63%). Benzaldehyde (25.99%) and benzeneethanol (17.11%) were the main constituents identified. Plant sample P-2 (female) and P-3 (male) were collected from the same place and they are just different in gender. Sample P-3 was given the local name ‘mekan endod’, which means it is the endod that can’t give seed or fruit. Thirty-seven and forty-two components were identified from the aromas of sample P-2 and P-3, which comprised 87.12% and 88.37% of the total aromas, respectively, and twenty-four components are same for these two samples. Aldehydes and ketones are predominant in both sample P-2 (78.26%) and P-3 (54.25%). Plant sample P-4 was also given the local name ‘mekan endod’, the same as sample P-3, but they differed in collection site. Forty-six components were identified from the aromas of sample P-4, which comprised 94.01% of the total aromas, and acetic acid (11.27%), benzyl alcohol (6.21%) and 3-methylbutanoic acid (5.31%) were the major constituents. Comparison between sample P-3 and P-4, which are same in gender but different in collection place, showed that only fourteen components were same in the two plant samples, which comprised 30% of the total forty-six compounds of aroma of sample P-4.

Sixty components in total were identified from the four essential oils, and only three compounds (phytone, pentacosane and hexadecanoic acid) were identical. This indicates that the four plant samples were widely different. Main constituents of essential oil of sample P-1 (Arebi endod) were phytol (21.56%) and hexadecanoic acid (23.87%), among other nineteen components comprising 64.61% of the total oil. Ten and twenty-two components were identified from the essential oils of sample P-2 (female) and P-3 (male), comprised 66.48% and 69.64% of the total essential oils, respectively, and only six components are same for these two plant samples. The major components of essential oils are phytone (21.23%) and hexadecanoic acid (20.07%) for sample P-2, and phytone (17.41%) and phytol (26.29%) for sample P-3. Thirty-six compounds, comprised 67.98% of the total essential oil, were detected from sample P-4, and main constituents of the essential oil of sample P-4 were (+)-spathulenol (9.07%) and hexadecanoic acid (13.54%).

The results of antimicrobial activity assay of the essential oils of four *P. dodecandra* samples were shown in Table 2. As seen in Table 2, no obvious antimicrobial activity was shown for all the four essential oils.

## 3. Discussion

The chemical composition of the four plant samples of *P. dodecandra* was quite different for both aromas and essential oils. Plant sample P-1 was given the local name ‘Arebi endod’, which indicates that it may be transferred from Arabiya, and may not native to Ethiopia [7]. Sample P-1 is monoecious, and its fruit is red and different from local *P. dodecandra* (sample P-2, green). From both aromas and essential oils, we can also find several common components between this nonnative plant and other three local plants, such as benzaldehyde, sulcatone, β-ionone, phytone and hexa- decanoic acid. Sample P-2 and P-3, collected in the same region, are different in gender, and their usages by local peoples are also different. The leaves of sample P-2 (femal) were widely used as soap, especially for making cotton white, and it was also used as molluscicide for the control of the snails that are the vectors of bilharzias [7,9,21], but sample P-3 (male) was used for animal abortions and rabies treatment by local peoples [9,22]. The chemical composition of these two samples is more similar than that of the other samples, and nearly 60% of the compounds are the same. Sample P-4 is also male, but different from sample P-3 in the collection place. Sample P-3 was collected around Addis Ababa, in the center of Ethiopia, and sample P-4 was collected around Lake Tana, in northwest Ethiopia. Between the two male samples (P-3 and P-4), fourteen compounds in aromas and nine compounds in essential oils are the same, which were less similar than sample P-2 and P-3.

The presence or absence of constituents in the aromas or essential oils composition is difficult to explain. Phytone, a dominant and behaviorally active component in male orchid bee fragrances [23], was found to be the only compound that existed in both aromas and essential oils of all four plant samples. 2-Tridecanone, 2-tetradecanone, β-ionone, phytone and phytol were detected in both the aroma and essential oil of sample P-3, and only phytone was identified from aroma and essential oil of sample P-2. One sesquiterpene, (+)-spathulenol, and other ten components were common in both the aroma and essential oil of sample P-4. The differences in chemical composition between the aromas and essential oils may be due to the different extraction methods (HS-SPME and steam distillation). Compared with steam distillation, HS-SPME is obviously better owing to more components being extracted, higher retrieval matching and sensitivity, and it can be used for the comparison between same plants of different place or plants from the same genus.

## 4. Materials and Methods 

Four samples of *P. dodecandra*, which were different in gender or location, were collected for this study in August 2017. The leaves of sample P-1 were collected in the garden of the Ethiopia Biodiversity Institute, Addis Ababa. The leaves of samples P-2 (female) and P-3 (male) were collected in Akaki Kality, Addis Ababa. The leaves of sample P-4 (male) were collected in the mountainous area of Emfraz, on the northeast shore of Lake Tana, Amhara. The four samples were taxonomically identified by Amare Seifu Assefa, a botanist from Ethiopian Biodiversity Institute. Four voucher specimens (PDB-2017-8, PDF-AAK-2017-8, PDM-AAK-2017-8 and PDM-AZ-2017-8) have been deposited in the Herbarium of Ethiopian Biodiversity Institute.

The leaves of all samples were coarsely powdered and transferred into a flask with 1.5 L water. Then it was subjected to a steam distillation process in a Clevenger’s apparatus for eight hours. The distillates were saturated with NaCl and extracted with diethyl ether. The organic phase was dried by anhydrous Na_2_SO_4_, and then recycled the organic solvent at 30 °C to give the oil. All the four oils were stored at 4 °C in refrigerator for further use.

The aromas of plant leaves were captured by solid-phase microextraction (SPME) using a 75 μm carboxen/polydimethylsiloxane (CAR/PDMS) fiber. For this purpose, powdered leaves were loaded into a 20 mL vial. After 40 min of exposure under 85 °C, the fiber containing the extracted aromas was then injected into the gas chromatography-mass spectrometry (GC-MS) and kept in the injector for 3 min under 250 °C.

GC-MS analysis of the oils and aromas were carried out using an Agilent gas chromatograph (model 6890, Agilent Technologies, Santa Clara, CA, USA), fitted with a HP-INNOWax capillary column (30 m × 0.25 mm × 0.25 μm). This chromatograph was coupled to a mass spectrometer-detector (model Agilent 5973N, Santa Clara, CA, USA). Gas chromatography condition: the temperature was programmed at 50 °C for the first three minutes, increased at a rate of 5 °C/minute to 180 °C, then at a rate of 10 °C/minute to 250 °C and held isothermal at 250 °C for the next ten minutes; The injector temperature was 250 °C; The injected volume was 1 μL for oil; Helium was used as carrier gas; Flow rate was 0.8 mL/min with a split ratio of 2:1 (aromas) or 10:1 (oils); Mass spectrometry condition: EI ionization mode, 70 eV, scan range 20–450 amu, ion source temperature was 230 °C, quadrupole mass spectrometer temperature was 150 °C. Individual components were identified by matching their mass spectra with those of the spectrometer data base (NIST, Gaithersburg, MD, USA; Wiley, Hoboken, NJ, USA). For quantification purposes, relative area percentages were used without the use of correction factors.

*Escherichia coli* (ATCC 25922), *Staphylococcus aureus* (ATCC 25923)*, Bacillus subtilis* (ATCC 6633) and *Candida albicans* (ATCC 10231) were used for antimicrobial evaluation. Antimicrobial activity assays were performed in 96-well sterilized microplates using a microdilution method described previously [24,25]. The 18-h-old bacterial cultures from *E. coli*, *S. aureus* and *B. subtilis* were added to LB broth medium (1 L water, 10 g tryptone, 5 g yeast extract, and 10 g NaCl) to reach 1 × 10^5^ CFU/mL, and the 4-day-old spores from *C. albicans* were added to PDB medium (potato 20%, glucose 2%) to reach 1 × 10^3^ spores/mL. The test samples were dissolved in DMSO, and their final concentrations were ranged from 0.5 to 512 μg/mL, which were determined by 2-fold serial dilution method. The wells containing test strains and diluted samples were incubated at 37 °C (24 h) for bacteria and 28 °C (4 days) for fungi. The wells containing a culture suspension and DMSO were run as negative controls. Kanamycin (for bacteria) and nystatin (for fungi) were introduced as positive controls. All experiments were repeated twice. The minimal inhibitory concentration (MIC) was defined as the lowest antibiotic concentration that produced complete growth inhibition of the tested microorganisms.

## 5. Conclusions

In the present study, we investigated the chemical composition of aromas and essential oils of four *P. dodecandra* samples which differed in collection place or gender, and a comparison of the essential oil and aroma components between the four plant samples was also performed. This analysis revealed that aldehydes and ketones, such as sulcatone, 2-nonanone, benzaldehyde and phytone, are the major components in the aromas. It also indicated that phytone, phytol and hexadecanoic acid were prominent in the essential oils. Such a difference in chemical composition is probably due to the different extraction methods used for the aromas and essential oils. It should also be mentioned that only six terpenes, which are usually prominent in most of the aromas or essential oils of other plants, were detected in the four *P. dodecandra* samples.

In conclusion, this is the first study on the essential oil and aroma of *P. dodecandra*, an important Ethiopian medicinal plant. Even if no distinct bioactivity was observed, the results of this research can also provide some references and basis for a better understanding and utilization of Ethiopian medicinal plants.

## Figures and Tables

**Table 1:** Chemical composition of the aromas and essential oils, from four *P. dodecandra* samples.

No.	Compounds	Sample P-1		Sample P-2		Sample P-3		Sample P-4	
		Aroma (%)	Oil (%)	Aroma (%)	Oil (%)	Aroma (%)	Oil (%)	Aroma (%)	Oil (%)
1	Dimethyl sulfide	0.67	―	0.10	―	―	―	―	―
2	2-Methyl-propanal	0.96	―	―	―	―	―	―	―
3	Acetone	―	―	0.46	―	―	―	―	―
4	2-Butanone	―	―	0.35	―	1.15	―	―	―
5	2-Methylbutanal	0.61	―	―	―	―	―	1.37	―
6	3-Methylbutanal	0.59	―	―	―	1.74	―	2.01	―
7	2-Ethylfuran	4.95	―	―	―	―	―	1.11	―
8	Pentanal	―	―	―	―	1.65	―	0.35	―
9	Dimethyl disulfide	―	―	0.45	―	0.78	―	―	―
10	Hexanal	0.91	―	0.37	―	―	―	2.46	―
11	Dimethyl trisulfide	―	0.56	―	―	―	―	―	―
12	1-Acetyl-1-cyclohexene	―	―	―	―	0.51	―	―	―
13	Pyridine	1.06	―	―	―	―	―	―	―
14	2-Heptanone	―	―	0.71	―	5.41	―	―	―
15	Heptanal	1.13	―	1.01	―	―	―	―	―
16	Sabinene	―	―	―	―	―	―	3.18	―
17	2-Methyl-1-butanol	2.19	―	―	―	―	―	―	―
18	2-Hexenal	3.97	―	1.40	―	―	―	2.95	―
19	2-Methylpyridine	―	―	―	―	1.61	―	―	―
20	Hexadecane	―	―	―	3.86	―	―	―	―
21	2-Pentyl-furan	1.38	―	1.33	―	7.72	―	―	―
22	1-Pentanol	0.76	―	―	―	―	―	―	―
23	2,3-Dimethylpyridine	―	―	―	―	2.03	―	―	―
24	3-Octanone	―	―	0.17	―	―	―	―	―
25	Styrene	―	―	―	―	7.55	―	―	―
26	Methylpyrazine	1.48	―	―	―	―	―	―	―
27	Para-cymene	―	―	―	―	―	―	4.76	―
28	2-Octanone	―	―	0.26	―	0.69	―	―	―
29	Hexanenitrile	―	―	―	―	1.78	―	―	―
30	2,5-Dimethylpyrazine	―	―	―	―	0.42	―	―	―
31	(*Z*)-6-Octen-2-one	―	―	1.71	―	―	―	―	―
32	Sulcatone	3.20	―	4.73	―	5.87	―	2.34	―
33	1-Hexanol	3.17	―	―	―	0.78	―	0.62	―
34	(*Z*)-3-Hexen-1-ol	―	―	―	―	―	―	1.80	―
35	2-Nonanone	1.80	―	30.80	―	1.97	―	―	―
36	Nonanal	2.07	―	―	1.09	2.10	―	―	―
37	(*Z*)-9-Methyl-2-undecene	―	―	1.55	―	―	―	―	―
38	2-Decanone	―	―	5.57	―	0.60	―	―	―
39	Acetic acid	4.92	―	―	4.47	―	―	11.27	―
40	2-Nonanol	―	―	1.59	―	―	―	―	―
41	Benzaldehyde	25.99	―	8.60	―	15.75	―	4.99	―
42	Nerolidol	―	0.92	―	―	―	―	―	1.32
43	1-Pentadecene	―	―	1.03	―	1.53	―	―	―
44	Linalool	―	―	―	―	―	―	0.82	―
45	2-Methylpropanoic acid	―	―	―	―	―	―	0.84	―
46	3,5-Octadien-2-one	―	―	2.11	―	―	―	―	―
47	(+)-Calarene	―	―	―	―	2.82	―	―	―
48	1,2-Propanediol	―	―	―	―	―	―	1.14	―
49	Isophorone	―	―	―	―	1.22	―	―	―
50	2-Undecanone	―	―	3.29	―	―	―	―	―
51	3,5-Dimethyl-1,2,4-trithiolane	―	―	―	―	―	―	―	0.79
52	Benzonitrile	―	―	0.41	―	1.79	―	―	―
53	γ-Butyrolactone	―	―	―	―	―	―	2.77	―
54	Acetophenone	1.29	―	3.59	―	4.53	―	―	―
55	2-Methoxy-4-vinylphenol	―	1.54	―	―	―	―	―	―
56	2-Dodecanone	―	―	0.74	―	―	―	―	―
57	Cryptone	―	―	―	―	―	―	―	0.47
58	3-Methylbutanoic acid	―	―	―	―	―	―	5.31	―
59	6,10-Dimethyl-2-undecanoe	―	―	0.36	―	0.50	―	―	―
60	4-Ketoisophorone	―	―	―	―	1.03	―	―	―
61	α-Terpinenyl acetate	―	―	―	―	―	―	―	1.09
62	Ethyl palmitate	―	0.61	―	―	―	―	―	―
63	γ-Hexalactone	2.82	―	―	―	―	―	0.75	―
64	Valencene	―	―	―	―	0.59	―	―	―
65	5-Phenyl-2-picoline	―	0.76	―	―	―	―	―	―
66	Isophytol	―	0.51	―	―	―	0.73	―	0.49
67	Benzyl acetate	―	―	―	―	―	―	0.35	―
68	Naphthalene	2.04	―	―	―	0.42	―	4.15	―
69	2-Hexen-4-olide	―	―	―	―	―	―	0.81	―
70	1,13-Tetradecadiene	―	―	0.61	―	―	―	―	―
71	6*E*,8*E*-Heptadecadiene	―	―	―	―	1.01	―	―	―
72	Eicosane	―	―	―	1.91	―	―	―	―
73	Tetracosane	―	0.82	―	―	―	0.53	―	―
74	2-Tridecanone	―	―	1.82	―	0.92	0.93	―	―
75	β-Dihydroionone	―	―	1.15	―	0.79	―	―	―
76	Hexanoic acid	0.76	―	―	―	―	―	3.95	―
77	Alpha-ionone	0.87	―	0.41	―	0.44	―	―	0.60
78	1-Methylnaphthalene	―	―	―	―	―	―	1.00	―
79	3-Methylsalicylic aldehyde	1.16	―	―	―	―	―	―	―
80	Geranyl acetone	―	―	1.71	―	0.36	―	0.49	0.60
81	2-Tetradecanone	―	―	0.87	―	0.54	0.82	―	―
82	Dodecanoic acid	―	2.67	―	―	―	―	―	0.96
83	Benzyl alcohol	―	―	0.67	―	0.85	―	6.21	0.40
84	Pentacosane	―	0.70	―	2.74	―	0.93	―	0.56
85	Benzeneethanol	17.11	―	―	―	―	―	3.57	0.57
86	Geranyl linalool	―	0.47	―	―	―	―	―	0.29
87	β-Ionone	3.24	―	2.65	―	1.09	0.53	1.77	1.65
88	1-Nonadecene	―	―	―	―	―	0.90	―	―
89	1-Octadecene	―	1.06	―	―	―	―	―	―
90	1-Hydroxy-7-methylbicyclo [4.2.1] non-7-en-2-one	―	―	―	―	1.39	―	―	―
91	(*E*)-3-Hexenoic acid	―	―	―	―	―	―	1.88	―
92	Hexacosane	―	―	―	3.20	―	0.70	―	―
93	(*E*)-2-Hexenoic acid	―	―	―	―	―	―	1.89	―
94	Caryophyllene oxide	―	―	―	―	―	―	―	0.77
95	β-Ionone epoxide	0.63	―	0.57	―	0.70	―	0.77	1.07
96	4-(2,2,6-Trimethylcyclohexa-1,3-dienyl)but-3-en-2-one	―	―	―	―	―	―	1.16	―
97	4,6-Dimethyl-1,2,3,5-tetra-thiacyclohexane	―	―	―	―	―	1.12	―	2.55
98	Phenol	―	―	―	―	―	―	0.60	―
99	2-Pentadecanone	―	―	―	―	―	1.86	―	―
100	Tetradecanoic acid	―	1.44	―	―	―	―	―	1.07
101	Octanoic acid	―	―	―	―	―	―	0.54	―
102	Phenanthrene	―	1.01	―	―	―	―	―	―
103	Heptacosane	―	―	―	5.11	―	1.12	―	―
104	2-Hexadecanone	―	―	―	―	―	2.13	―	―
105	4-Isopropylbenzyl alcohol	―	―	―	―	―	―	―	0.70
106	Nonacosane	―	―	―	2.80	―	1.83	―	―
107	(+)-Spathulenol	―	―	―	―	―	―	0.55	9.07
108	Phytone	1.21	3.04	2.85	21.23	3.30	17.41	1.11	3.95
109	4,7,9-Megastigmatrien-3-one	―	―	―	―	―	―	0.39	―
110	Nonanoic acid	―	―	―	―	―	―	0.53	0.79
111	4-Vinylguaiacol	―	―	―	―	―	―	―	3.61
112	4,6,8-Megastigmatrien-3-one	―	―	―	―	―	―	1.42	―
113	Carvacrol	―	―	―	―	―	―	―	2.06
114	6,10,14-Trimethyl-2-penta-decanol	―	―	―	―	―	1.19	―	―
115	Decanoic acid	―	―	―	―	―	―	―	0.60
116	Methylethylmaleimide	―	―	―	―	0.45	―	2.74	―
117	Glycerol	―	―	―	―	―	―	1.19	―
118	Dihydroactinidiolide	0.98	―	0.81	―	1.38	―	3.29	2.91
119	Farnesyl acetone	―	―	―	―	―	2.71	―	2.18
120	4-Vinylphenol	―	―	―	―	―	―	―	1.44
121	Benzoic acid	―	1.38	―	―	―	―	0.71	―
122	Indole	―	0.61	―	―	―	1.11	―	0.71
123	Neophytadiene	―	―	―	―	―	―	―	0.28
124	Benzeneacetic acid	―	―	―	―	―	―	0.34	―
125	Methyl linolenate	―	―	―	―	―	―	―	0.63
126	Vanillin	―	―	―	―	―	―	―	0.50
127	Phytol	―	21.56	0.31	―	0.61	26.29	0.74	4.11
128	Benzyl benzoate	―	―	―	―	―	1.61	―	―
129	Octacosane	―	―	―	―	―	1.93	―	―
130	Hexadecanoic acid	―	23.87	―	20.07	―	1.49	1.02	13.54
131	Triacontane	―	―	―	―	―	1.77	―	―
132	Oleic acid	―	1.06	―	―	―	―	―	1.26
133	Linoleic acid	―	―	―	―	―	―	―	1.25
134	Methyl-11,14,17-eicosa-trienoate	―	―	―	―	―	―	―	3.14
**Compounds in Total**		**30**	**19**	**37**	**10**	**42**	**22**	**46**	**36**

**Table 2:** Antimicrobial activities of the essential oils, from four *P. dodecandra* samples (MIC: µg/mL).

Essential Oil	*E. Coli*	*S. Aureus*	*B. Subtilis*	*C. Albicans*
Plant sample P-1	64	64	>128	>128
Plant sample P-2	>128	>128	>128	>128
Plant sample P-3	>128	>128	>128	>128
Plant sample P-4	>128	>128	>128	>128
Kanamycin	8	1	1	―
Nystatin	―	―	―	4
